# Hypothermia predicts mortality in critically ill elderly patients with sepsis

**DOI:** 10.1186/1471-2318-10-70

**Published:** 2010-09-27

**Authors:** Ravindranath Tiruvoipati, Kevin Ong, Himangsu Gangopadhyay, Subhash Arora, Ian Carney, John Botha

**Affiliations:** 1 Department of Intensive Care medicine, Frankston Hospital, Frankston, Victoria, 3199, Australia

## Abstract

**Background:**

Advanced age is one of the factors that increase mortality in intensive care. Sepsis and multi-organ failure are likely to further increase mortality in elderly patients.

We compared the characteristics and outcomes of septic elderly patients (> 65 years) with younger patients (≤ 65 years) and identified factors during the first 24 hours of presentation that could predict mortality in elderly patients.

**Methods:**

This study was conducted in a Level III intensive care unit with a case mix of medical and surgical patients excluding cardiac and neurosurgical patients.

We performed a retrospective review of all septic patients admitted to our ICU between July 2004 and May 2007. In addition to demographics and co-morbidities, physiological and laboratory variables were analysed to identify early predictors of mortality in elderly patients with sepsis.

**Results:**

Of 175 patients admitted with sepsis, 108 were older than 65 years. Elderly patients differed from younger patients with regard to sex, temperature (37.2°C VS 37.8°C p < 0.01), heart rate, systolic blood pressure, pH, HCO_3_, potassium, urea, creatinine, APACHE III and SAPS II. The ICU and hospital mortality was significantly higher in elderly patients (10.6% Vs 23.14% (p = 0.04) and 19.4 Vs 35.1 (p = 0.02) respectively). Elderly patients who died in hospital had a significant difference in pH, HCO_3_, mean blood pressure, potassium, albumin, organs failed, lactate, APACHE III and SAPS II compared to the elderly patients who survived while the mean age and co-morbidities were comparable. Logistic regression analysis identified temperature (OR [per degree centigrade decrease] 0.51; 95% CI 0.306- 0.854; p = 0.010) and SAPS II (OR [per point increase]: 1.12; 95% CI 1.016-1.235; p = 0.02) during the first 24 hours of admission to independently predict increased hospital mortality in elderly patients.

**Conclusions:**

The mortality in elderly patients with sepsis is higher than the younger patients. Temperature (hypothermia) and SAPS II scores during the first 24 hours of presentation independently predict hospital mortality.

## Background

The elderly population is increasing in several countries across the world [1]. It is estimated that at least for the next 25 years the elderly population is expected to increase more rapidly than any other age group [2]. The use of intensive care resources increases with age and it is estimated that half of all intensive care unit (ICU) days are currently occupied by patients older than 65 years of age[1]. This trend is likely to continue in the future with a greater number of elderly patients accounting for intensive care admissions and utilising a greater proportion of heath care resources. It has been demonstrated in several studies that advanced age is one of the factors that increases mortality in intensive care [3,4]. While age was shown to be strongly associated with mortality, there is evidence to suggest that acute physiological impairment, reduced functional reserve, patient preferences for care, atypical presentations, differences in physician practices and associated co-morbidities could all be factors that increase mortality rather than age perse [5,6]. Elderly patients frequently suffer from one or more severe chronic illnesses before hospitalisation and are less able to meet the physiological demands of critical illness. Studies comparing outcomes between younger patients and elderly patients show mortality and morbidity increase significantly with advancing age [3].

Sepsis remains an important cause for admission to intensive care units accounting for over 25% of all ICU admissions [7]. It is known to be the leading cause of death in non coronary intensive care units [8] and the mortality remains high in spite of the recent advances in the management of patients with sepsis [9]. The incidence of sepsis appears to increase with increasing age because of the associated increase in co-morbidities, malnutrition, institutionalisation, exposure to instrumentation and altered immune function [10]. Furthermore studies reveal the mortality of elderly patents with sepsis to be greater than in younger patients [4,3]. There are no studies to our knowledge identifying the predictors of mortality in elderly patients with sepsis admitted to intensive care units.

While there are several causes for a high mortality in elderly patients with sepsis [11], one of the contributing factors could be a delay in identifying elderly septic patients who are critically ill. The strategy of early identification and institution of appropriate management that was proven to improve the outcome in patients with sepsis [12], was also shown to improve outcome in the elderly patients [13]. Given the limited physiological reserve of the elderly patients, early identification and the use of appropriate interventions is probably more relevant in this group of patients. In this study of septic patients admitted to our ICU we aimed to compare the characteristics and outcomes of elderly patients (> 65 years) versus younger patients (</= 65 years) and identify early (< 24 hours) predictors of mortality in elderly patients with sepsis.

## Methods

Our hospital's Human Research Ethics Committee have reviewed the study and waived formal ethical application as the study is a retrospective audit of data routinely collected for patient care and not experimental research. This study is a retrospective observational study over a 3 year period (July 2004 and May 2007) including all septic patients admitted to our intensive care unit (ICU). We have followed the definitions according to 2001 SCCM/ESICM/ACCP/ATS/SIS International Sepsis Definitions Conference in identifying and classifying patients with sepsis [14]. Accordingly we defined

*Sepsis: *Clinical syndrome defined by the presence of both infection and a systemic inflammatory response.

*Severe sepsis: *Sepsis complicated by organ dysfunction

*Septic shock: *Acute circulatory failure characterized by persistent arterial hypotension unexplained by other causes.

Data was collected from our ICU database (STATIC), our hospital's pathology database and the case records of the patients included in the study. Data on physiological, laboratory variables and scores derived from the scoring systems, [Acute physiology age and chronic health evaluation III score (APACHE III) and simplified acute physiology score II (SAPS II)] during the first 24 hours of presentation with sepsis were collected and the most abnormal values during the first 24 hours of presentation were analysed. The physiological variables included age, PaO_2_, PaCO_2_, pH, HCO_3_, heart rate, blood pressure (systolic, diastolic and mean), respiratory rate, temperature and organs failed during the first 24 hours of admission to ICU. The biochemical variables analysed were serum sodium, potassium, blood glucose, urea, creatinine, bilirubin, albumin, hematocrit, white blood cell count, C reactive protein and lactate. The primary outcomes of interest were ICU and in-hospital mortality

While there is no clear definition of "elderly" in the medical literature, most of the studies classified patients older than 65 years as elderly[3-5]. Hence we defined elderly patients to be more than 65 years.

### Statistical analysis

The relationship between age group and demographic, physiological and laboratory variables and scores was assessed using Fisher's exact test in categorically-scaled data or using Student's t-tests or Mann-Whitney U tests (as appropriate) for continuously-scaled data. The relationship between and within age groups and selected demographic, physiological and laboratory variables and scores with that of mortality was estimated using logistic regression analysis. The incidence rate of ICU and in-hospital mortality was calculated by dividing the number of deaths within each outcome by the number of person-days of observation. Finally, the comparison between age groups of the time to ICU or in-hospital death or discharge as assessed by the logrank test applied to Kaplan-Meier curves. Cox proportional hazards regression models were constructed to estimate the magnitude of the force of mortality from ICU or in-hospital deaths by age group, after adjustment of potential confounders found to be associated with the outcome in crude analyses. All results are presented with 95% confidence intervals. Statistical significance was deemed to have been attained when the two-tailed p-value was less than 0.05. Data analysis was undertaken using SPSS 15.0 (SPSS Inc, Chicago, IL) and Stata/MP 11.0 (StataCorp LP, College Station TX).

## Results

Between July 2004 and May 2007 a total of 1,965 patients were admitted to our ICU of which 175 patients had sepsis. Of these patients 121 (69.1%) were admitted with medical problems and 54 (30.9%) following surgical interventions. Almost all patients (97.1%) were admitted as emergency admissions. Of the 175 septic patients 18.3% had sepsis; 81.7% had severe sepsis of which 44% had septic shock. The source of sepsis was pulmonary in 56 (32%), urinary tract in 37 (21.1%), abdominal in 17 (9.7%), blood in 16 (9.1%), cutaneous/soft tissue in 7 (4%), and other sources (cardiac, neuro, bone, ENT, gynaecological) in 34 (19.4%). In 8 (4.6%) patients the source of sepsis was not clear. *Sputum culture *was positive in 80 patients with Candida as the commonest organism (43 patients). Other organism included methicillin resistant staphylococcus (MRSA) (8 patients) Pseudomonas species, (6 patients), Klebsiella species (5 patients) and other organisms (18 patients). *Blood culture *was positive in 50 patients. E coli was isolated in 11 patients, methicillin sensitive staphylococcus (MSSA) in 7 patients, coagulase negative staphylococcus in 7 patients, Klebsiella in 5, MRSA in 5, Streptococcus in 4 and other organisms in 11 patients. *Urine culture *was positive in 25 patients with Candida in 12 patients, E coli in 6 patients, enterococcus in 4 and other organisms in 3 patients. The overall ICU and hospital mortality were 18.3% and 29.1% respectively.

Of the 175 patients, 108 (61.7%) patients were older than 65 years. The demographics, co-morbidities, physiological and laboratory variables and scores of all patients at the time of admission ICU are presented in table 1. As shown in the table elderly patients had a trend towards an increased incidence of cardiac co-morbidities and a statistically significant proportion of these patients were males. The elderly patients had a lower bicarbonate, pH and, temperature. They had higher systolic blood pressure, serum potassium, urea, creatinine, APACHE III and SAPS II scores that were statistically significant.

**Table 1:** Comparison of characteristics between elderly and younger patients

*Variable*	*</= 65 years (n = 67)*	*> 65 years (n = 108)*	*P Value*
Median age (IQR)	56 (46-60)	76 (71-79)	<0.01

Sex (M:F)	27:40	66:42	<0.01

*Co-morbidities*			

Chronic renal failure	11	13	0.15**

Chronic obstructive pulmonary disease	13	25	0.83**

Congestive cardiac failure/Ischemic heart disease/Hypertension	27	69	0.05**

Diabetes mellitus	9	20	0.66**

*Physiological variables*	Median (IQR)	Median (IQR)	

PaO_2 _(torr)	93 (67-126)	101 (73.7-172.7)	0.25

PaCO_2_(torr)	41 (34-50)	39 (32-49)	0.59

HCO_3 _(mmol/L)*	20.56 (4.09)	17.97 (5.38)	<0.01

pH	7.32 (7.25-7.41)	7.29 (7.15-7.37)	0.02

Heart rate	110 (99-128)	105 (88.5-120)	0.02

Systolic blood pressure (mmHg)	120 (95.5-141)	138 (113.5-152)	<0.01

Diastolic blood pressure (mmHg)	65 (51-72)	62 (50-74)	0.77

Mean blood pressure(mmHg)	84 (67-95)	87 (77-98)	0.13

Temperature (degree Celsius)	37.8 (37-38.5)	37.2 (36.5-37.9)	<0.01

Respiratory rate	26 (18.5-32.5)	26 (20-30)	0.28

Number of organs failed on admission	1 (0-2)	1 (0-2)	0.10

*Laboratory variables*			

Sodium (mmol/L)	142 (138.2-145.7)	142 (139-145)	0.71

Potassium (mmol/L)	4.25 (0.84)	4.50 (0.78)	0.04

Urea (mmol/L)	9 (5.2-15.8)	14.5 (9.2-22.5)	<0.01

Creatinine (umol/L)	80.5 (51.7-147.5)	160 (100-250)	<0.01

Bilirubin (umol/L)	14 (8.25-27)	15.5 (8-27.5)	0.93

Albumin (g/L)	28.85 (7.39)	29.27 (5.87)	0.69

Blood sugar (mmol/L)	7.8 (6.1-12.2)	9.4 (6.7-12.2)	0.23

C reactive protein (mg/L)	197.6 (91.4-274.3)	168.7 (62.3-253.5)	0.28

Lactate (mmol/L)	2.1 (1.4-4.3)	2.8 (1.5-6.1)	0.29

White cell count (×10^9/L)	16.2 (11.3-20.9)	15.2 (10.2-24)	0.98

Hematocrit (%)	0.30 (0.26-0.33)	0.31(0.27-0.35)	0.10

Positive culture in the first 24 hours (%)	40.3	44.6	0.62

*Scores*			

APACHE III	57 (41.2-70.2)	77 (58.5-93)	<0.01

APACHE III without age component	48 (36-65)	57.5 (40.2-77.7)	0.03

SAPS II	34.5 (26.7-42)	47 (39-56.5)	<0.01

SAPS II without age component	27 (20-34)	31 (22-41.5)	0.01

• * Mean (SD), student t test

• ** Fisher's exact test

• All other analyses was by Mann-Whiney test

• IQR: inter-quartile range

The actual and predicted mortalities of all patients based on SAPS II and APACHE III scores showed that the actual mortality and predicted mortality were comparable (actual mortality 29.1%, SAPS II predicted mortality 35.9% (p = 0.36) and APACHE III predicted mortality 32.4% (p = 0.75)). This was similar with patients aged </= 65 years (Actual mortality 19.4%, SAPS II predicted mortality 24.3% (p = 0.49) and APACHE III Predicted mortality 22.8% (p = 0.60)) or >65 years (Actual mortality 35.2%, SAPS II predicted mortality 43.3% (p = 0.31) and APACHE III Predicted mortality 38.4% (p = 0.76)). Further when SAPS II and APACHE III scores were analysed without the age component it was noted that there were significantly different between older and younger patients suggesting that the physiological derangements were more severe in older patients (table 1).

**Figure 1:**
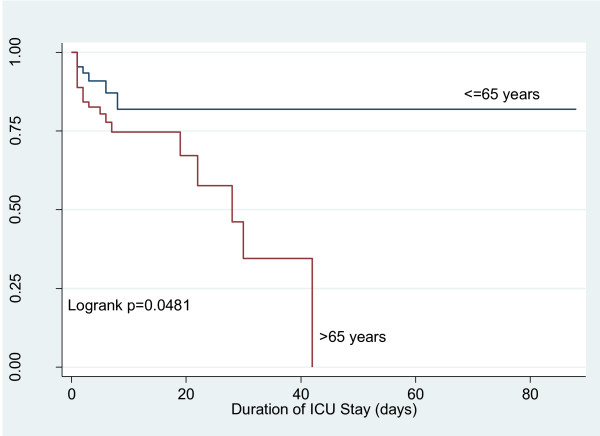
**Kaplan-Meier curve of the survival function for ICU**.

**Figure 2:**
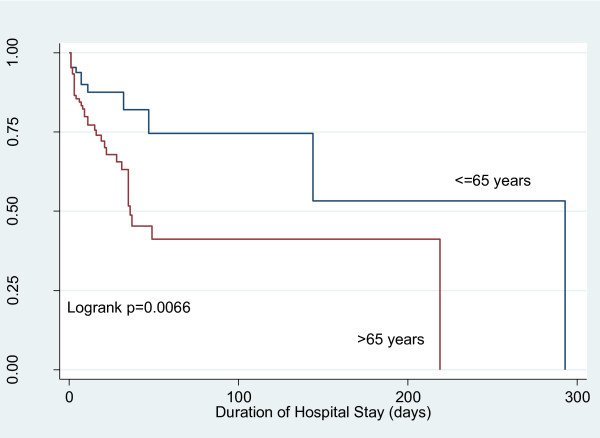
**Kaplan-Meier curve of the survival function in-hospital mortality**.

The comparison of ICU and hospital mortality between elderly and younger patients is presented in table 2. As shown in table 2 the crude mortality rates in ICU and hospital are higher in elderly patients. Elderly patients had 2.70 (95% CI 1.14, 7.41) times the rate of ICU mortality and 2.89 (95% CI 1.50, 5.91) times the rate of in-hospital mortality compared to younger patients. Similar results were found in assessments of Kaplan-Meier curves (Figure 1 and 2). After adjustment for disease severity, elderly patients showed increased risk of in-hospital mortality (Hazard Ratio (HR) = 2.21; 95% CI 1.04, 4.71) compared to younger patients. While the adjusted risk was increased in elderly patients for the outcome of ICU mortality, it failed to reach statistical significance (HR = 1.50; 95% CI 0.60, 3.76). The number of patients discharged to nursing home or chronic hospital were comparable between younger and elderly patients (10 Vs 29 patients respectively; p = 0.54).

**Table 2:** Comparison of outcomes between elderly and younger patients

*Outcome*	*</= 65 years (n = 67)*	*> 65 years (n = 108)*	*P Value*
ICU mortality, rate per 1000 person-days	15.4	41.8	0.01**

Hospital mortality, rate per 1000 person-days	5.0	14.5	< 0.001**

• ** mid-p exact test

When comparing elderly patients who died in the hospital to those who were discharged, there was no significant difference in mean age, sex or the co-morbidities. Patients who died had lower mean blood pressure, pH, bicarbonate, albumin and higher potassium, lactate, APACHE III and SAPS II scores and a greater number of organs failed (table 3).

**Table 3:** Comparison of elderly patients who died in hospital and survived to hospital discharge

*Variable*	*Died in hospital* *Mean (SD) (n = 38)*	*Survived to hospital discharge Mean (SD) (n = 70)*	*P Value*
Median age (IQR)	75 (70-78.2)	76 (71-80.5)	0.63

Sex (M:F)	19:19	45:23	0.14**

*Co-morbidities*			

Chronic renal failure (%)	14.7	14.5	1.00**

Chronic obstructive pulmonary disease (%)	38.2	21.8	0.14**

Congestive cardiac failure/Ischemic heart disease/Hypertension (%)	67.6	77.1	0.33**

Diabetes mellitus (%)	18.7	24.5	0.60**

*Physiological variables*	*Median (IQR)*	*Median (IQR)*	

PaO_2 _(torr)	113 (73.5 -195)	99. (75-167)	0.31

PaCO_2 _(torr)	39 (31.5-57)	39 (33-45)	0.21

HCO_3_(mmol/L)*	15.41 (6.45)	19.56 (4.02)	< 0.01

pH	7.15 (6.99-7.31)	7.31 (7.23-7.38)	< 0.01

Heart rate	110 (90-127.5)	105 (86-114)	0.11

Systolic blood pressure (mmHg)	132 (105-147)	140 (120-154)	0.12

Diastolic blood pressure(mmHg)	60 (50-68)	65 (50-75.2)	0.10

Mean blood pressure(mmHg)	82 (72-92.5)	89.5 (80.7-99)	0.04

Temperature (degree Celsius)	36.7 (35.4-37.9)	37.3 (36.8-37.9)	0.06

Respiratory rate	24 (15.5-32)	26 (21.7-30)	0.60

Number of organs failed	2 (1-3)	1 (0-1)	< 0.01

*Laboratory variables*			

Sodium (mmol/L)	143 (140-145)	141 (138-144)	0.14

Potassium (mmol/L)	4.78 (0.92)	4.33 (0.64)	0.01

Urea (mmol/L)	16.4 (9.7-23.9)	12.6 (8.1-19.7)	0.16

Creatinine (umol/L)	185 (130-250)	130 (90-241)	0.05

Bilirubin (umol/L)	18 (9-35)	14 (8-26)	0.47

Albumin (g/L)	27.29 (7.49)	30.25 (4.60)	0.04

Blood sugar (mmol/L)	9.3 (6.7-14.3)	9.6 (6.5-11.5)	0.55

C reactive protein (mg/L)	163 (78.5-248.1)	168.7 (61-254.1)	0.87

Lactate (mmol/L)	4 (2-10.1)	2.2 (1.3-4.0)	< 0.01

White cell count (×10^9/L)	14.5 (7.2-23)	15.4 (11.3-24)	0.21

Hematocrit (%)	0.31 (0.27-0.36)	0.32 (0.27-35)	0.90

Positive culture in the first 24 hours (%)	51.4	38.5	0.28**

*Scores*			

APACHE III	91 (76.5-114.5)	65 (53.7 -83)	< 0.01

SAPS II	57 (45.5-70)	43.5 (37-50)	< 0.01

*Outcome*			

Duration of ICU stay (days; Mean (SD))	2 (1-6.2)	2 (1-6)	0.97

Duration of hospital stay (days; Mean (SD))	9 (3-32)	15 (8-31.5)	0.09

• * Mean (SD), student t test

• ** Fisher's exact test

• All other analyses was by Mann-Whiney test

• IQR: inter-quartile range

Logistic regression of the variables identified SAPS II (OR [per point increase]: 1.12; 95% CI 1.016-1.235; p = 0.02) and temperature (OR [per degree centigrade decrease] 0.51; 95% CI 0.306- 0.854; p = 0.010) to be the independent predictors of increased hospital mortality in elderly patients. In younger patients SAPS 2 score independently predicted mortality ((OR [per point increase]: 1.07; 95% CI 1.00 to 1.16; p = 0.04). When all the patients (elderly and younger) were included the predictors were the SAPS2 score ((OR [per point increase]: 1.07; 95% CI 1.001 to 1.155; p = 0.03) and temperature (OR [per degree centigrade decrease] 0.58; 95% CI 0.399-0.872; p = 0.008).

## Discussion

The results of this study suggest that the mortality in elderly patients was higher than that of the younger patients. The elderly patients however had no statistically significant differences in terms of co-morbidities other than a trend towards an increased incidence of cardiovascular co-morbidities that did not quite reach statistical significance. They differed significantly in terms of physiological variables (pH, HCO3, Temperature, systolic blood pressure) and the admission APACHE III and SAPS II scores. The data on elderly patients who died and those who survived to hospital discharge were comparable in terms of age and co-morbidities. However, they had significant differences in pH, HCO_3_, lactate, albumin, potassium, number of organs failed and the APACHE III and SAPS II scores. Of the variables analysed, temperature and SAPS II were independent predictors of hospital mortality in elderly patients.

The overall mortality of all patients and elderly patients admitted with sepsis to our ICU is comparable to other published results [3,15,16]. The higher mortality in elderly patients in our study was probably related to higher initially severity of the illness rather than the higher incidence of co-morbidities. These findings differ from some of the other studies comparing older and younger patients with sepsis[3,4]. The possible reason for this could be that most of the patients admitted to our ICU with sepsis are older and a relatively smaller proportion of patients were younger (about 1.7% of patients were younger than 30 years, 6.3% were younger than 40 years and 13.7% were younger than 50 years of age). Indeed the mean age of all the patients admitted to our ICU with sepsis is 66 years and only 38% of the patients were younger than 65 years.

In our study there was no statistically significant difference in the discharge to nursing home or other chronic care facilities between elderly and younger patients. Other studies [8] have noted a significant difference. The exact reason for this may not be identified given the retrospective nature of our study. However it is possible that during the study period there was an over representation of elderly patients admitted to our ICU with little co morbidity. This in turn may have resulted in an outcome in elderly patients comparable to younger patients. Furthermore patients with a poor quality of life and from the chronic care facilities may not have been admitted to ICU due to limitations of care imposed by the patients (refusing admission to ICU, intubation and ventilation etc) themselves and/or their relatives.

While there are several studies identifying the predictors of mortality in patients with sepsis, there are no studies identifying predictors of mortality specifically in critically ill elderly patients with sepsis. The results of our study suggest that temperature and SAPS II during the first 24 hours of presentation to be independent predictors of mortality.

Fever is known to be an important feature of sepsis and was thought to be an adaptive response to aid in defence of the invading organisms. However, the exact role of temperature in influencing the outcome of sepsis is still unclear. Some experimental studies suggest that induced hypothermia may have a beneficial effect by reducing energy requirement and activating cell-protecting pathways [17]. The clinical studies however suggest inability to mount a febrile response to be associated with increased mortality in patients with sepsis [18-20]. Clemmer et al evaluated the consequences of clinical hypothermia associated with severe sepsis and septic shock. In their study patients with hypothermia had a higher frequency of central nervous system dysfunction (88% vs. 60%), increased serum bilirubin concentration (35% vs. 15%), prolonged prothrombin times (50% vs. 23%), shock (94% vs. 61%), failure to recover from shock (66% vs. 26%), and death (62% vs. 26%) [19].

Our study shows hypothermia to be an independent predictor of mortality in elderly patients with sepsis. Experimental data suggests that preventing or early correction of hypothermia by rewarming in sepsis was associated with improved outcomes [21,22]. Wangg et al in their rat model of sepsis induced by lipopolysaccharide found that when normothermia was maintained the lung injury was alleviated as compared with the rats that became hypothermic. Xiao and Remick have demonstrated from their studies on mice that warming significantly increased the peripheral blood cell count, including the neutrophils, suggesting that warming could augment innate immunity and improve survival [21]. Whether correcting hypothermia (by active rewarming) in elderly sepsis patients will improve mortality remains to be evaluated.

The efficacy of SAPS II and APACHE III scores to predict hospital mortality in elderly patients has not been validated [23]. However it is interesting to note that in our study SAPS II was a predictor of mortality and APACHE III was not. The reason for this could be that the predictive power of APACHE III system is based on physiological conditions as well as the underlying co morbidities and age. Knaus et al showed that 73.1% of the mortality prediction power in the APACHE III system was due to physiological conditions, with the underlying disease contributing to 13.6% and age to only 7.3% [24]. SAPS II on the other hand is primarily based on the physiological variables and does not include co morbidities in estimating the mortality. In addition, some of the variables included in our study such as serum bicarbonate and potassium which differed significantly between survivors and non survivors were part of the SAPS II and not APACHE III score.

Organ failure and lactate in sepsis are known predictors of mortality in patients with sepsis [25,26]. While there was a statistically significant difference in the number of organs failed and the lactate concentration between survivors and non survivors (table 3), neither of the two were independent predictors of mortality. It is possible that we have used the worst value in the first 24 hours and this may not have a predictive value for mortality. The study by Vosylius et al showed that the discriminatory power of organ dysfunction on day 1 was poor and evolving organ dysfunction following admission to the ICU strongly affected the outcome [27]. Nguyen et al in their study on patients with severe sepsis and septic shock showed the clearance of lactate early in the hospital course to be a predictor of mortality rather than a single reading of lactate [28].

The hospital mortality in our patients is related to the severity of acute physiological impairment at the presentation. These findings are similar to other studies reporting outcome in elderly patients admitted to the intensive care units [29-31]. These findings suggest that the increased mortality in elderly patients is likely to be the significant derangement in the physiology. The beneficial effects of early management of severe sepsis are well known [12,13]. Early identification of elderly patients at high risk and appropriate management of acute physiological derangements may further improve the outcomes. The impact of such management may be more obvious given the diminished physiological reserve in elderly patients [10].

## Conclusions

Elderly patients with sepsis had a higher mortality when compared to younger patients. SAPS II and temperature were independent predictors of mortality in elderly patients with sepsis.

## Abbreviations

ACCP: American College of Chest Physicians; APACHE: Acute physiology age and chronic health evaluation; ATS: American Thoracic Society; ESICM: European Society of Intensive Care Medicine; ICU: Intensive care unit; OR: Odds ratio; SAPS: Simplified acute physiology score; SCCM: Society of Critical Care Medicine; SIS: Surgical Infection Society

## Competing interests (Financial or non financial)

The authors declare that they have no competing interests.

## Authors' contributions

RT: Conception and design, acquisition of data, analysis and interpretation of data, drafting the manuscript and final approval of the version to be published

KO: Acquisition of data, design of study, revising manuscript critically for important intellectual content and final approval of the version to be published

HG: Revising manuscript critically for important intellectual content and final approval of the version to be published

SA: Revising manuscript critically for important intellectual content and final approval of the version to be published

IC: Revising manuscript critically for important intellectual content and final approval of the version to be published

JB: Drafting the manuscript, revising manuscript critically for important intellectual content, overall supervision and final approval of the version to be published

## Pre-publication history

The pre-publication history for this paper can be accessed here:

http://www.biomedcentral.com/1471-2318/10/70/prepub
